# miR-18a expression correlates with ATM and p53 levels and poor prognosis in lymphomas

**DOI:** 10.7150/jca.83854

**Published:** 2023-05-08

**Authors:** Hao Zhou, Yuxiang He, Chunlin Ou, Xiaoyun He, Pengfei Cao

**Affiliations:** 1 Department of Hematology, Xiangya Hospital, Central South University, Changsha 410008, Hunan, China.; 2 Department of Oncology, Xiangya Hospital, Central South University, Changsha 410008, Hunan, China.; 3 Department of Pathology, Xiangya Hospital, Central South University, Changsha 410008, Hunan, China.; 4 Departments of Ultrasound Imaging, Xiangya Hospital, Central South University, Changsha 410008, Hunan, China.; 5 National Clinical Research Center for Geriatric Disorders, Xiangya Hospital, Central South University, Changsha 410008, Hunan, China.

**Keywords:** miR-18a, Epstein-Barr virus, Lymphoma, Genomic instability, Biomarker

## Abstract

microRNAs (miRNAs) are non-coding, endogenous, small-molecule RNAs. They are involved in cell proliferation, differentiation, apoptosis, and metabolism. Additionally, they play an essential role in the development and progression of various malignancies. Recent research has revealed that miR-18a plays an important role in cancer development. However, its role in lymphoma is not yet fully understood. In this study, we investigated the clinicopathological characteristics and potential functional roles of miR-18a in lymphomas. First, we predicted the potential downstream genes of miR-18a using miRTarBase software and subjected these downstream genes to Gene Ontology (GO) and Kyoto Encyclopedia of Genes and Genomes (KEGG) analyses to determine the potential mechanisms of action of these genes. We found that these target genes were closely related to cellular senescence, the p53 signaling pathway, and other signaling pathways. From the predicted downstream target genes, ATM and p53 were selected as the target genes; their deletion in patients with lymphoma was detected using the fluorescence *in situ* hybridization technique. The results showed that some patients with lymphoma have a deletion of the *ATM* and *p53* genes. In addition, the deletion rates of *ATM* and *p53* were positively correlated with the expression of miR-18a. Next, the expression levels of miR-18a and the deletion rates of *ATM* and *p53* were used for correlation and prognostic analyses with patient clinical information. The findings revealed a significant difference in disease-free survival (DFS) between patients with lymphoma with *ATM* deletion and those with a normal *ATM* gene expression (*p* < 0.001). Moreover, a significant difference in overall survival (OS) and DFS between patients with *p53* deletion and those with normal *p53* expression was observed (*p* < 0.001). The results indicate that the deletion of *ATM* and *p53* downstream of miR-18a is closely associated with the development of lymphoma. Thus, these biomarkers may serve as key prognostic biomarkers for lymphomas.

## Introduction

Lymphoma is a malignant tumor that originates from the lymphopoietic system. The occurrence and development of lymphomas are complex processes involving multiple steps, stages, and factors 1. Both environmental (such as physical, chemical, and biological) and host factors play essential roles in lymphoma development 2,3. Pathogenic infections such as those caused by the Epstein-Barr virus (EBV) also play an important role in the development of lymphomas 4. Therefore, complex interactions between environmental factors and host genetic susceptibility influence the occurrence and development of lymphomas.

Genomic instability has been found in most lymphomas using various molecular biological assays 5, 6. Among these genomic instabilities, chromosomal instability is the most common manifestation in malignant tumors; genomic instability causes tumor cells to gain a growth advantage 7, 8. The genotype of lymphoma is highly complex. Amplification of the 13q31 locus, which encodes the miR-17-92 cluster, is often observed in B-cell lymphomas. Moreover, members of the miR-17-92 cluster are overexpressed and amplified in lymphoma 9. Our previous study found a significant correlation between the expression of miR-18a, a member of the miR-17-92 cluster, and the reactivation of EBV due to DNA damage 10. However, the role of miR-18a in the development of lymphoma has not been elucidated. The aim of this study was to investigate the relationship between miR-18a interaction with its downstream target genes and genomic instability in lymphomas. In addition, we explored the clinical significance of these target genes in lymphoma development.

## Materials and methods

### Tissue samples and clinical data

The study included 100 patients who were diagnosed with lymphoma and 23 patients with inflammatory lymph nodes as the control group. All patients included in this study were diagnosed between January 2008 and December 2015 at the Xiangya Hospital, Central South University, China. The diagnosis of all patients with lymphoma was confirmed by clinical presentation, imaging, biochemistry, immunology, bone marrow morphology, and lymphoma biopsy. The sample included 59 cases of non-Hodgkin's B-cell lymphoma, 34 cases of NK/T-cell lymphoma, and 7 cases of Hodgkin's lymphoma. Clinical cases were classified based on sex, age, lymph node infiltration, *ATM* deletion, and *p53* deletion with high *miR-18a* expression. All lymphomas were diagnosed and staged in accordance with the National Guidelines for the Treatment of Malignant Lymphoma in China 11. Treatment effects were observed after 3-6 courses of conventional treatment for lymphoma. Patients were informed about the purpose of the study and signed informed consent. The study was approved by the ethical review committee of Xiangya Hospital, Central South University. Patient characteristics for the 100 lymphoma cases are presented in **Table 1**.

#### *In situ* hybridization

After dewaxing and hydrating the pathological sections, the slides were transferred to 3% H_2_O_2_ and protease buffer to inactivate the endogenous enzymes. The slides were then treated with pepsin diluted in 3% citric acid. After pepsin digestion, the slides were fixed with a fixative at room temperature for 10 min. The slides were hybridized to the probe overnight at 59 °C. Subsequently, the slides were rinsed and placed in a blocking solution at 37 °C for 30 min. Biotinylated murine anti-digoxin was added dropwise to the slides, incubated for 120 min at room temperature, and then washed with PBST buffer. The SABC was added dropwise, incubated for 30 min, and washed repeatedly with PBST buffer. Finally, the sections were developed using DAB color development rinses and hematoxylin re-staining. Following dehydration and mounting, sections were observed and imaged under a microscope (OLYMPUSBX-51, Japan). Two pathologists independently determined the *in situ* hybridization scores of the tissue samples.

#### Fluorescence *in situ* hybridization (FISH)

To prepare the lymph node tissue sections for analysis, they were first placed in an oven for 30 min at a temperature of 80 °C. Next, the sections were immersed in a preheated dewaxing agent at 68 °C for 15 min. After dewaxing, slides were washed twice for 5 min in 100% ethanol. Then, they were immersed in a permeabilizer for 20 min at 90 °C, followed by a 3 min wash with preheated deionized water at 37 °C. Subsequently, the slides underwent digestion through immersion in a preheated protease solution at 37 °C for a duration between 10 and 40 min. Following digestion, the tissue sections underwent two 5 min rinses with a washing solution. Gradient dehydration of the slides was accomplished through sequential immersion in 70%, 85%, and 100% ethanol concentrations for 2 min each, followed by air drying at room temperature. After dehydration, 10 μl of denatured probe mixture was injected onto each slide, and they were immediately covered with cover slips. For co-denaturation, the slides were exposed in a hybridizer at 85 °C for 5 min. Lastly, the slides were placed in a clean wet box at 42 °C overnight. After hybridization, the slides were placed in a pre-warmed 0.4× SSC/0.3% NP-40 solution at 67 °C for 30 s, and then dried at room temperature. The dried slides were re-stained with 15 µL DAPI and covered with a cover slip. Finally, the slides were observed under a fluorescence microscope by selecting the appropriate filter set. Two probes were used in this study - ATM and p53 probes, located at 14q32 and 17p13.1, respectively (all purchased from Beijing Golden Bodega Company, China). In normal interphase cells, the ATM probe showed two red signals and the p53 probe showed two green signals. To obtain 10 normal control specimens, 200 cells were selected under an oil microscope to observe the fluorescence signal and establish the threshold value. This threshold value was based on the results of the 23 control patients with lymph node inflammation. Two hundred cells from each member of the control group and from each patient were observed to calculate the average number of fluorescent particles in each cell. A fluorescent particle count of less than the threshold value (4-5 particles/cell) was considered negative. Patients with specimen fluorescence greater than the threshold were considered positive, whereas those below the threshold were considered negative.

### Prediction and functional analysis of target genes

MiRTarbase is an experimentally validated miRNA-target interaction database (http://mirtarbase.mbc.nctu.edu.tw/php/index.php) containing 4076 miRNAs and 23054 target genes based on experimental evidence (including reporter analysis, western blotting, microarray, or pSILAC). The database was used to construct and visualize miRNA-mRNA regulatory networks. GO and KEGG pathway analyses were performed using the online analysis website CancerMIRNome (http://bioinfo.jialab-ucr.org/CancerMIRNome/) to functionally annotate potential target genes.

### Statistical analysis

The SPSS 26.0 statistical package was used for statistical analysis. Survival data were obtained using Kaplan-Meier analysis. The log-rank test was used to determine the differences between the survival curves. Pearson χ2-test was used to test the correlation between miR-18 expression and *ATM* and *p53* gene deletions. A *p* value of less than 0.05 was considered significant.

## Results

### The expression level of miR-18a in patients with lymphoma

In a previous study, we detected the expression level of miR-18a using *in situ* hybridization and found that its expression level was increased in EBV-positive patients with lymphoma. This finding suggested that *miR-18a* expression was positively correlated with EBV infection. Further analysis revealed that *miR-18a* was highly expressed in more than half of the patients with lymphoma, with a positivity rate of 51%. In contrast, the positivity rate was only 4.6% in patients with inflammatory lymph nodes (**Table 1**). We found that the positivity rate and index of *miR-8a* were significantly higher in lymphoma specimens than in control lymphadenitis specimens (*p* < 0.01).

### Analysis of potential downstream genes of miR-18a in lymphoma

In a previous study, we found that miR-18a plays a role in EBV-associated lymphoma by inhibiting the DNA damage response and promoting EBV-associated genomic instability 10. To further investigate the potential downstream genes of miR-18a, we used miRTarBase software to predict the potential downstream genes of miR-18a (**Supplementary Table 1**). The downstream genes were also subjected to GO and KEGG analyses to explore their potential mechanisms of action. We found that these genes were closely related to signaling sets such as cellular senescence and the p53 signaling pathway. Based on the above analyses, we selected *ATM* and *p53* among the *miR-18a* downstream target genes for further study.

### Detecting the deletion rate of *ATM* and *p53* genes in lymphomas

**Figure 2:**
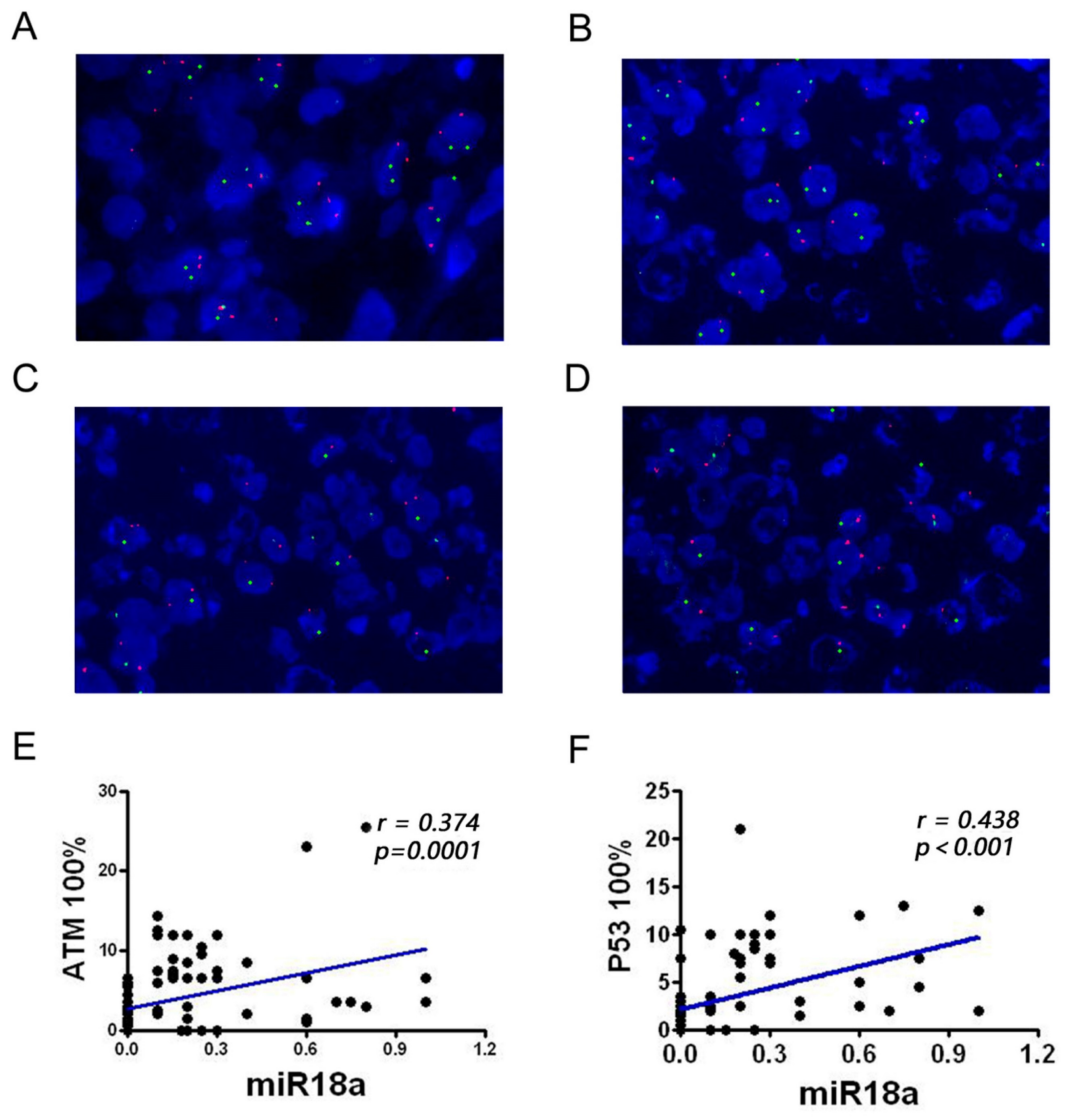
Detection of the *ATM* and *p53* genes in pathological sections from patients with lymphoma, observed via FISH technique, and analysis of miR-18a and their correlation. (A) Normal *ATM* and *p53* genes; (B) *ATM* gene deletion; (C) *p53* gene deletion; (D) Simultaneous *ATM* and *p53* gene deletions. (E-F) Correlation analysis of miR-18a expression with* ATM* or *p53* deletion rates (magnification 1000 ×).

The deletion rates of *ATM* and *p53* genes associated with genomic instability were examined in 100 patients with lymphoma and 23 control inflammatory specimens using FISH. The results showed that the *ATM* and *p53* deletion rates were significantly higher in patients with lymphoma than in lymph node inflammatory specimens (*P* < 0.01). The two red and two green dots in **Figure 2A** indicate that both *ATM* and *p53* are normally expressed. One red and two green lines in **Figure 2B** indicate that the *ATM* gene is absent and the *p53* gene is normally expressed. Two reds and one green in **Figure 2C** indicate that the *ATM* gene is normal and the *p53* gene is absent. One red and one green in **Figure 2D** indicate that the *ATM* gene is absent, and the *p53* gene is absent.

### Correlation analysis of miR-18a expression with p53 and ATM deletion rate

To further investigate the correlation between *miRNA-18a* and genomic instability in patients with lymphoma, we performed a correlation analysis of miR-18a expression with *ATM* and *p53* deletion rate (**Table 1**, **Figure 2E and 2F**). *miR-18a* expression was positively correlated with *ATM* (r = 0.374, *p* = 0.001) and *p53* deletion rates (r = 0.438, *p* < 0.001). These results also suggest that *miR-18a* expression levels are higher in patients with lymphoma and the molecular basis of lymphoma is associated with genomic instability.

### Correlation between miR-18a expression and survival prognosis of patients with lymphoma

**Figure 3:**
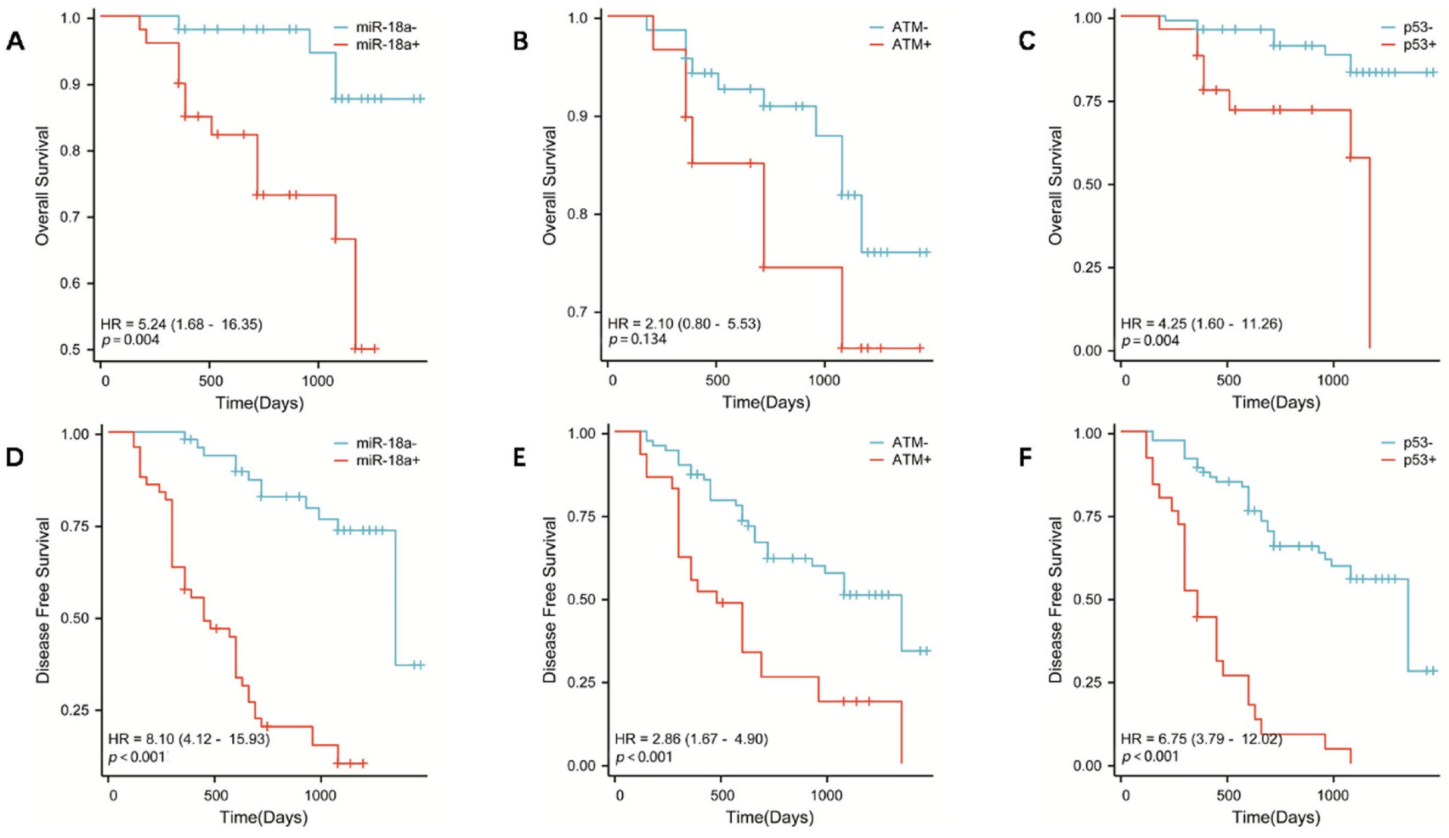
OS in patients with lymphoma with miR-18a positive expression, *ATM* gene deletion, or *p53* gene deletion. (A, B, C, respectively) DFS in patients with lymphoma with miR-18a-positive expression, *ATM* gene deletion, or *p53* gene deletion. (D, E, F, respectively)

To further investigate the correlation between *miR-18a* expression and survival prognosis of patients with lymphoma, we collected clinical prognostic information from patients through regular and long-term telephone follow-up. The collected follow-up clinical data were statistically analyzed, and survival curves were plotted using SPSS 26.0. The results showed that the OS was lower in patients with miR-18a-positive expression of lymphoma than in patients with *miR-18a*-negative expression (*p* = 0.004) (**Figure 3A**). Moreover, DFS was lower in miR-18a-positive-expressing patients than in miR-18a-negative-expressing patients (*p* < 0.001) (**Figure 3D**). DFS in patients with *ATM* deletion was significantly different from DFS in those with normal *ATM* genes (*p* < 0.001) (**Figure 3E**). However, only patients with *ATM* deletion showed a trend towards worse OS (*p* = 0.128), compared with those with normal *ATM* genes (**Figure 3B**). Both OS and DFS in patients with *p53* deletion were significantly different from those in patients with normal *p53* (*p* = 0.004 and *p* < 0.001 for OS and DFS, respectively) (**Figure 3 C and 3F**).

## Discussion

miRNAs are endogenous non-coding RNAs with an average length of approximately 22 nucleotides, and they have been found to regulate gene expression at the post-transcriptional level 12, 13. They are widely present in nematodes, Drosophila, plants, and mammals 14-16. Highly evolutionarily conserved miRNAs are fully or incompletely paired with the 3'-untranslated region (UTR) of target gene mRNAs, thereby inhibiting their translation. Subsequently, they exert physiological effects that regulate cell proliferation, differentiation, and individual development 17,18. In addition, miRNAs play crucial roles in the occurrence and development of various diseases.

A growing body of evidence has highlighted the vital role of miRNAs in cancer progression 19-21. The miR-17-92 cluster is one of the best-characterized novel non-coding RNA clusters 22. It consists of seven members: miR-17-5p, miR-17-3p, miR-18a, miR-19a, miR-20a, miR-19b, and miR-92. This cluster of miRNAs is aberrantly amplified in lymphomas and other malignancies 23-26. Upregulation of this gene cluster promotes lymphangiogenesis, especially in B-cell lymphomas. For example, overexpression of miR-17-92 has pro-oncogenic effects by promoting proliferation and decreasing apoptosis in mantle cell lymphoma tumor cells 27, 28. Robaina *et al*. 29 found that miR-17 upregulation led to reduced OS in childhood Burkitt's lymphoma and was associated with a lack of expression of the pro-apoptotic gene BIM. In addition, Fassina *et al*. 30 reported that miR-17-92 was significantly overexpressed in germinal center-type diffuse large B-cell lymphoma and could be used as a reliable and standardized diagnostic tool for the subclassification of large B-cell lymphoid-like neoplasms. As the most representative miRNA in the miR-17-92 cluster, miR-18a plays a pro-tumorigenic role in a variety of malignancies. Our previous study demonstrated that miR-18a is highly expressed in EBV-positive lymphomas and mediates the growth of lymphoma cells by inducing EBV reactivation, which could promote malignancy of EBV-associated lymphomas. However, little is known regarding the role of miR-18a in lymphoma development. In this study, we further analyzed the data and found miR-18a overexpression and a significantly higher positivity rate and positivity index in lymphoma compared to that in lymph node inflammatory control specimens (*p* < 0.01).

*ATM* and *p53* are target genes that are closely associated with cellular senescence and the p53 signaling pathway. Activation of *ATM*, *p53*, and the DNA damage response (DDR) is an important mechanism to stop the proliferation of genomically altered cells. *ATM* is located on chromosome 11q22.3, with a full length of 184 kb and contains 66 exons. The coding region of the gene is flanked by variant 3′ and 5′-UTRs. These variants may be associated with the post-transcriptional regulation of ATM levels under physiological conditions 31. *ATM* encodes a nuclear phosphoprotein, which is a member of the phosphatidylinositol 3-kinase (PI3K) family 32. PI3K family members have been implicated in DNA repair after damage and cell cycle regulation. *p53* is also an important anti-oncogene, commonly known as the "molecular police" in DNA damage and repair, which can play an important role in promoting apoptosis of cancer cells and preventing cellular carcinogenesis. *p53* also plays an essential role in the repair of cellular DNA following damage 33. Previous studies have reported that both *ATM* and *p53* play important roles in lymphoma development. For example, Zhou *et al*. 34 found that activation of the ATM-Chk2-p53-p21 pathway blocked cell cycle progression and induced apoptosis in NK/T-cell lymphoma cell lines. Bhalla *et al*. 35 reported that *ATM* knockout induced mitochondrial deacetylase SIRT3 activity and disrupted the mitochondrial structure, thereby promoting the growth of diffuse large B-cell lymphoma. Based on these findings, ATM and p53 are two proteins that work closely together and may be involved in the construction of anti-cancer barriers. miRNAs play an important role in controlling gene expression by inhibiting protein translation or promoting messenger RNA degradation. miRTarBase software predicted potential miR-18a downstream target genes, which were then analyzed for GO and KEGG enrichment. The results indicated that miR-18a is closely associated with cellular senescence, the p53 signaling pathway, and other signaling pathways. We propose that miR-18a is likely to induce malignancy in lymphoma by affecting the expression of *ATM* and *p53* genes.

Furthermore, the disruption of the ATM/p53 pathway can impact the synthesis of the corresponding proteins, which ultimately affects the survival and prognosis of individuals with lymphoma. Massive cell lymphoma (MCL) is a particularly aggressive subtype of lymphoma that has been linked to the deletion of *ATM* and *p53*; *ATM* has been identified as the most frequently mutated gene in MCL 36. Interestingly, ATM inactivation did not significantly affect the survival of patients with MCL, whereas TP53 mutations had a substantial negative impact on OS in MCL 37. Nevertheless, testing for *ATM* mutations remains important. Previous studies have demonstrated that cells with defective *ATM* function exhibit heightened radiosensitivity, which may be advantageous for treating highly chemo resistant lymphoma subtypes with radiotherapy 38. In addition, chronic lymphocytic leukemia cells with *ATM* mutations have shown increased resistance to doxorubicin, likely due to their inability to activate the pro-apoptotic p53 pathway following drug administration 39. It has been suggested that the deletion of the ATM/p53 pathway may be a common selection mechanism in malignant B lymphocytes 37. In the present study, we utilized the FISH technique to investigate the frequency of *ATM* and *p53* gene deletions in lymphoma. FISH is a molecular genetic technique that has been recently used in a wide range of clinical applications. It can directly reveal the relationship between DNA sequences in the nucleus or chromosomes of specific cells by *in situ* hybridization of labeled probes of specific molecules to chromosomes and the development of fluorescent color 40. The basic principle of the FISH technique is qualitative localization and relative quantitative analysis of the nucleic acid targets in a specimen using a nucleic acid probe directly or indirectly labeled with fluorescein, based on the principle of base complementarity. The FISH technique overcomes limitations of traditional cytogenetic approaches by using sequence-specific probes to rapidly and accurately reveal structural abnormalities in chromosome number, identify chromosome origin, and analyze complex karyotypes. Owing to the disadvantages of poor chromosome morphology and difficulty in observation, numerous studies have shown high sensitivity and specificity of the FISH technique to examine multiple leukemia fusion genes and other site-specific gene deletions or mutations 41. Currently, FISH technology is widely used for the detection of genes at the molecular level in China and abroad. In this study, FISH was used to detect the deletion rate of the genomic instability-related molecules *ATM* and *p53*. The data revealed that the rate of *ATM* and *p53* gene deletion was significantly higher in patients with lymphoma than in inflamed lymph node tissues (*P* < 0.01). Combined with the analysis of clinical data, we found that the deletion of *ATM* and *p53* correlated with the clinical grade of lymphoma and extra-nodal metastases, independently of age and sex. Moreover, the overall survival of patients with *ATM* and *p53* deletions was significantly shorter. Correlation analysis of miR-18a expression with *ATM* and *p53* deletion rates showed that miR-18a expression was positively correlated with *ATM* deletion rates (r = 0.374, *p* = 0.001) and *p53* deletion rates (r = 0.438, *p* < 0.001). Based on the findings, it appears that miR-18a is strongly linked to the expression of *ATM* and *p53* genes. Therefore, we propose that miR-18a may act as a regulator of ATM and p53, ultimately impacting the prognosis of individuals with lymphoma.

## Conclusion

In conclusion, this study revealed that miR-18a is an upregulated biomarker of lymphoma and its expression level is positively correlated with the deletion of the *ATM* and *p53* genes. miR-18a is likely to induce malignant behavior in lymphoma by targeting the downstream genes *ATM* and *p53*, which are associated with poor prognosis in lymphoma. It should be noted, however, that while we have established a clear association between miR-18a and lymphoma in clinical samples, we did not explore the relationship between individual subtypes and molecules. Thus, further *in vitro* and *in vivo* experiments on the pathogenesis of miR-18a are required to verify the role of its regulated molecular network in lymphoma.

## Supplementary Material

Supplementary table.

## Figures and Tables

**Figure 1:**
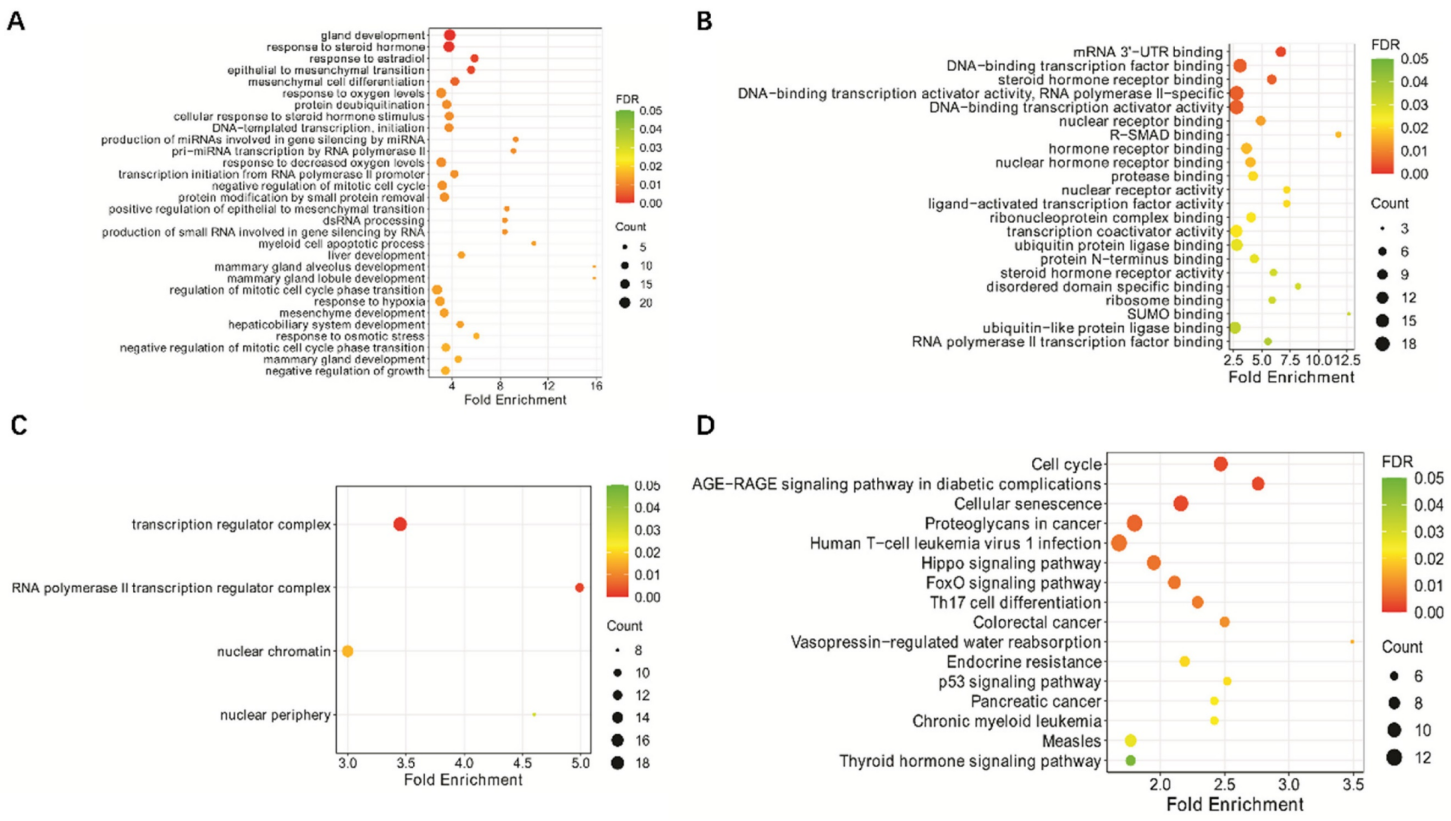
KEGG pathway enrichment analysis and GO functional enrichment analysis of hsa-miR-18a predicted target genes: (A) GO biological process; (B) GO molecular function; (C) GO cellular component; (D) KEGG analysis.

**Table 1:** Expression levels of miR-18a in patients with lymphoma and lymphadenitis

	Lymphoma	Lymphadenitis	*p* Value
miR-18a ^High^	51	1	< 0.01
miR-18a ^Low^	49	22	

**Table 2:** Relationship between miR-18a expression and lymphoma clinicopathology and gene deletion characteristics

Clinical and pathological features	N	*miR-18a* +high	*miR-18a* -low	*p* Value
**Gender**				
Male	65	35	30	
Female	35	16	19	0.5304
**Age**				
≥50	55	28	27	1.000
<50	45	23	22	
**Extranodal lymph node metastasis**				
Yes	71	43	28	
No	29	8	21	0.0039
***ATM***				
+	63	39	24	
-	37	12	25	0.0037
***p53***				
+	51	32	19	
-	49	19	30	0.0018

## Abbreviations

miRNA: microRNA
FISH: fluorescence *in situ* hybridization
GC-DLBCL: germinal central diffuse large B-cell lymphoma
DFS: disease-free survival time
OS: overall survival time
GO: Gene Ontology
KEGG: Kyoto Encyclopedia of Genes and Genomes
PI3K: phosphatidylinositol 3-kinase
DDR: DNA damage response
UTR: untranslated region
MCL: massive cell lymphoma
